# Determination of optimal fiducial marker across image‐guided radiation therapy (IGRT) modalities: visibility and artifact analysis of gold, carbon, and polymer fiducial markers

**DOI:** 10.1120/jacmp.v13i5.3976

**Published:** 2012-09-06

**Authors:** Lydia L. Handsfield, Ning J. Yue, Jinghao Zhou, Ting Chen, Sharad Goyal

**Affiliations:** ^1^ Department of Radiation Oncology UMDNJ – Robert Wood Johnson Medical School & The Cancer Institute of New Jersey New Brunswick NJ USA

**Keywords:** fiducial marker, image‐guided radiation therapy (IGRT), tomotherapy, computed tomography (CT), kilovoltage (kV)

## Abstract

The purpose of this study was to evaluate the visibility and artifact created by gold, carbon, and polymer fiducial markers in a simple phantom across computed tomography (CT), kilovoltage (kV), and megavoltage (MV) linear accelerator imaging and MV tomotherapy imaging. Three types of fiducial markers (gold, carbon, and polymer) were investigated for their visibility and artifacts in images acquired with various modalities and with different imaging parameters (kV, mAs, slice thickness). The imaging modalities include kV CT, 2D linac‐based kilovoltage and megavoltage X‐ray imaging systems, kV cone‐beam CT, and normal and fine tomotherapy imaging. The images were acquired on a phantom constructed using Superflab bolus in which markers of each type were inserted into the center layer. The visibility and artifacts produced by each marker were assessed qualitatively and quantitatively. All tested markers could be identified clearly on the acquired CT and linac‐based kV images; gold markers demonstrated the highest contrast. On the CT images, gold markers produced a significant artifact, while no artifacts were observed with polymer markers. Only gold markers were visible when using linac‐based MV and tomotherapy imaging. For linac‐based kV images, the contrast increased with kV and mAs values for all the markers, with the gold being the most pronounced. On CT images, the contrast increased with kV for the gold markers, while decreasing for the polymer and carbon marker. With the bolus phantom used, we found that when kV imaging‐based treatment verification equipment is available, polymer and carbon markers may be the preferred choice for target localization and patient treatment positioning verification due to less image artifacts. If MV imaging will be the sole modality for positioning verification, it may be necessary to use gold markers despite the artifacts they create on the simulation CT images.

PACS number: 87

## I. INTRODUCTION

Inter‐ and intrafractional target motion has been observed in radiation oncology treatment therapy of various disease sites.^1^, ^4^ The motion can be attributed to physiological internal organ movements, patient setup uncertainties, and patient breathing. It is essential to account for these motion deviations to achieve desired dose coverage of the target volumes and minimize normal tissue toxicities. Correcting for target motion and location can allow the oncologist to prescribe tighter treatment margins around the tumor, possibly reducing the dose to normal tissue.^5^, ^7^

Image‐guided radiotherapy (IGRT) has been increasingly used in the radiation treatment of cancer patients to assist with correcting inter‐ and intrafractional motion. Various techniques of IGRT have been explored including transabdominal ultrasound,^8^, ^9^ implanted fiducial markers with in‐room MV or kV X‐rays,^4^, ^8^ optical surface tracking systems,^(10)^ implanted electromagnetic markers,^9^, ^11^ and in‐room CT‐based systems such as kVCT on rail, kilovoltage or megavoltage cone‐beam CT (CBCT) and helical MVCT. Many of these IGRT methods rely on comparing daily X‐ray images taken at the time of treatment to images created at the time of treatment planning. The target geometric deviation is then determined by aligning the soft tissue structures, bony anatomy,^(11)^ implanted fiducial markers,^4^, ^7^ or other landmarks to the corresponding ones on the original images. When X‐ray‐based imaging is used for IGRT, it is typically at the discretion of the oncologist or radiation therapist to align the daily images with the original treatment planning images, sometimes with the help of computer‐assisted registration software.^(10)^ Ideally, the alignment should be based on imaged target volumes and other anatomical structures. Due to suboptimal image quality, anatomical changes, or organ deformation of soft tissue target volumes and other tissue structures acquired at the time of treatment, the alignment process can be subjective, possibly leading to inaccurate patient setup.

Fiducial markers have been used in radiotherapy to assist with the registration process.^4^, ^7^, ^8^, ^12^ Normally, fiducial markers are placed inside or adjacent to the target volumes prior to the radiotherapy simulation and serve as surrogates to the target volumes. It is hoped that the fiducial markers can be clearly and easily identified and localized on both simulation and verification images. The utilization of the fiducial markers as surrogates of the target volumes can not only reduce the uncertainty introduced by human subjective judgment during the alignment process, but also expedite the process. A major prerequisite of using fiducial markers for this purpose is clear identification of the markers on the images without introducing a significant amount of artifacts. The reduction of artifacts on the planning CT is especially desirable, since extensive artifacts could interfere with structure delineation and dose calculations, especially if an inhomogeneity correction treatment planning algorithm is used.^13^, ^19^ Given the plethora of imaging systems available for positioning verification, questions remain about the optimal choice of fiducial marker material for any given imaging system and the effects of various imaging parameters on image quality. Adjusting imaging parameters such as kVp, mAs, and slice thickness may have different degrees of impact on the image quality for different fiducial makers.

To address these unknowns and to provide guidelines on the selection of an optimal type of fiducial makers for a given imaging modality, this study was designed to evaluate the impacts of fiducial markers constructed of different materials on the image quality of several commonly used imaging modalities.

## II. MATERIALS AND METHODS

### A. Fiducial marker materials, imaging modalities, and experimental setup

Cylindrical fiducial markers made of gold, carbon, and polymer (CIVCO Medical Solutions, Kalona, Iowa) were evaluated. These types of fiducial markers have been used clinically or are being approved for clinical use. The diameters and lengths of the gold, carbon, and polymer markers are 0.9 and 3 mm, 1 and 3 mm, 1 and 5 mm, respectively. The evaluated imaging modalities include a GE LightSpeed 16 CT simulator (GE, Waukesha, WI), a linac‐based on‐board imaging (OBI) system (Varian Medical Systems, Palo Alto, CA), a linac‐based MV portal imaging system (Varian Medical Systems, Palo Alto, CA), and a MV 3D imaging system from a TomoTherapy Hi•Art treatment system (TomoTherapy, Inc., Madison, WI). A simple $30\times 31\times 15\;{\text{cm}}^{3}$ phantom was constructed using 2 cm thick layers of Superflab bolus (Radiation Products Design, Inc, Albertville, MN). The near‐constant density of the Superflab phantom allowed us to quantify the artifacts introduced purely by the fiducial markers without the inconsistencies and artifacts of patient anatomy. Three of each type of the tested fiducial markers were inserted into the central layer of the phantom and were placed about 5 cm apart from each other. The phantom was then imaged under the following conditions: 3D CT imaging with the GE CT simulator (80, 100, and 140 kV with 1.25 mm and 2.5 mm slice thicknesses; 0.625, 1.25, 2.5, 3.75, and 5 mm slice thicknesses with 120 kV); 2D kV imaging with the linac‐based OBI system (60, 70, 75, 80, 90, 100, 110, and 120 kVp with 200 mAs; 50, 63, 80, 100, 125, 160, and 200 mAs with 80 kVp); 3D linac‐based OBI kV cone‐beam CT (60, 90, 100, and 125 kV); 2D MV X‐ray imaging (1 and 2 MU) using the linac‐based MV portal imager; and the normal and fine 3D MV imaging with the TomoTherapy Hi•Art system.

### B. Assessments

Qualitative and quantitative assessments of the visibility and artifacts produced by each marker were performed. To determine the visibility of each marker, a 15 cm by 15 cm area profile was drawn around each marker using the Eclipse treatment planning system's off‐line review image analysis tools (Varian Medical Systems, Inc, Palo Alto, CA), and the statistical information within that area was used to calculate the contrast‐to‐noise ratio. The contrast‐to‐noise ratio (CNR) is defined as, where is the maximum signal intensity produced by the markers, is the average background signal intensity, and is the standard deviation of the background noise. The maximum signal intensity was used to insure that the most visible part of each marker was recorded and analyzed. To quantitatively analyze the artifacts created by each marker, the standard deviation of the pixel values along a ring of increasing radius around each marker on a 2D image was computed. One thousand rings with radiuses ranging from 1 to 15 mm around each seed were used.

## III. RESULTS

### A. kV CT simulation imaging

**Figure 1:**
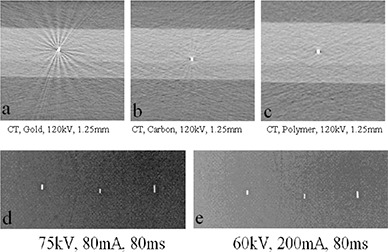
Markers under kV imaging: (a) CT, gold marker, 120 kV, 1.25 mm slice; (b) CT, carbon marker, 120 kV, 1.25 mm slice; (c) CT, polymer marker, 120 kV, 1.25 mm slice; (d) gold, carbon, and polymer markers, respectively, as seen under linac‐based kV imaging 75 kV, 80 mA, 80 ms; (e) gold, carbon, and polymer markers, respectively, as seen under linac‐based kV imaging 60 kV, 200 mA, 80 ms.

**Figure 2:**
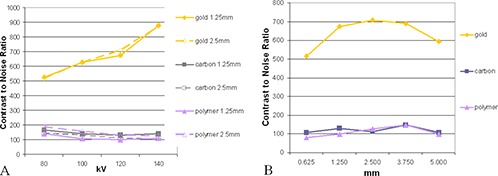
CT contrast‐to‐noise‐ratio (a) with varying kV, 1.25 mm (solid lines) and 2.5 mm (dashed lines) slice thicknesses; gold, carbon, and polymer markers are represented by diamonds, squares, and triangles, respectively. CT contrast‐to‐noise‐ratio (b) with varying slice thickness for gold, carbon, and polymer fiducial markers. Voltage was held at 120 kV.

(Figures 1a)–(c) display the fiducial markers imaged with the kV CT simulator. All tested markers could be observed clearly under the kV CT imaging without any ambiguity, and each material produced various degrees of artifacts. The gold markers ((Fig. 1a) had a significantly higher contrast‐to‐noise ratio, indicating a higher level of visibility. (Figure 2a) shows the dependence of the contrast (C) on kVp which exhibited different patterns for the gold markers verses the carbon and polymer markers. The contrast increased with increasing kV for the gold marker, but decreased slightly with increasing kV for the carbon and polymer markers. (Figure 2b) shows the dependence of the contrast on the CT slice thickness at 120 kVp. As the slice thickness increased from 0.625 mm to 5 mm, the contrast remained almost unchanged for the polymer and carbon markers. For the gold markers, the contrast increased with increasing slice thickness by almost 40% up to 2.5 mm slices. From 2.5 mm to 5 mm slice thickness, the contrast for the gold markers started to decrease with the increase of the slice thickness. At 5 mm of slice thickness, the decrease was about 15% compared to the peak contrast value.

**Figure 3:**
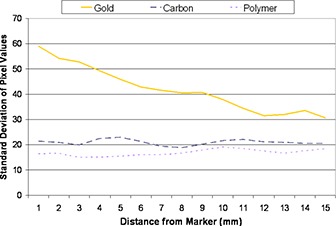
Variations of the pixel values of rings of increasing radius around each marker for a 120 kV, 1.25 mm slice CT image.

The pixel value distributions were measured for 1000 rings with radii ranging from 1 to 15 mm around each seed on the CT images acquired at 120 kVp and 1.25 mm slice thickness, and their standard deviations were computed for each ring. Figure 3 shows the values of the standard deviations along each ring with increasing ring radius. The gold marker presented a much larger standard deviation of pixel values than the carbon and polymer markers, indicating the production of a significant amount of artifact.

### B. Linac‐based 2D kV, 2D MV, kV CBCT, and MV helical imaging

**Figure 4:**
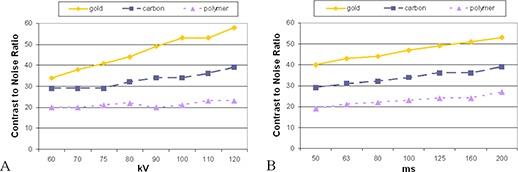
Change in contrast‐to‐noise‐ratio (a) with varying kV values for gold, carbon, and polymer fiducial markers; 200 mA, 80 ms held constant. Change in contrast‐to‐noise‐ratio (b) with varying exposure time for gold, carbon, and polymer fiducial markers; 200 mA, 80 kV held constant.

**Figure 5:**
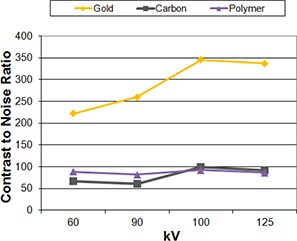
Linac‐based on‐board imaging kV cone‐beam CT contrast‐to‐noise ratio varying with kV; 80 mA held constant. Gold, carbon, and polymer markers are represented by diamonds, squares, and triangles, respectively.

(Figures 1d)–(e) show that all of the tested markers could be identified clearly under the linac‐based 2D kV imaging. None of the markers produced artifacts that would hinder their localization. For the kV images, gold demonstrated the highest contrast. The contrast increased with increasing kVp ((Fig. 4a) for all the markers regardless of material, with gold markers showing the most pronounced increase. Increasing the mAs ((Fig. 4b) increased each of the markers contrast‐to‐noise ratios almost linearly, with gold still demonstrating the highest CNR of the three markers. Figure 5 shows the effect of increasing the kV during cone‐beam CT acquisition. For gold markers, the contrast increased with increasing kV up to 100 kV, then decreased slightly. The carbon and polymer markers did not show a large change in contrast with increasing kV.

**Figure 6:**
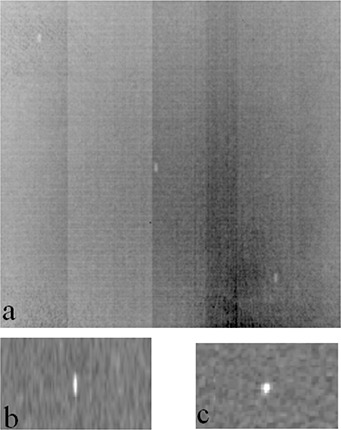
Markers under MV imaging: (a) linac‐based 2D MV image, 2MU (only the gold fiducial markers are visible); (b) tomotherapy MVCT normal scan, coronal view and (c) transverse view (only the gold fiducial markers are visible).

Only the gold fiducial markers were visible on the linac‐based 2D MV images ((Fig. 6a) and MVCT tomotherapy images ((Figs. 6b)–(c)). Slight blurring was observed in the coronal view of the tomotherapy MVCT image, but overall the gold markers did not produce significant artifacts under the MV imaging. The average contrast‐to‐noise ratio for the linac‐based 2D MV image was 6. For the tomotherapy fine and normal MVCT scans, the average CNR were 23 and 18, respectively.

## IV. DISCUSSION

The decision on which type of fiducial marker to use for the purpose of treatment positioning verification should be made based on the marker's visibility in both simulation CT and verification images, as well as the potential for dose perturbation during proton treatments.^9^, ^20^ To avoid these negative impacts of gold markers during treatment delivery, fiducial markers made of other materials such as carbon, polymer, and stainless steel have been introduced (CIVCO Medical Solutions, Kalona, IA).

Comparing three types of fiducial markers, our study showed that the contrast of gold markers was highly dependent on CT slice thickness and significantly increased with the increase of X‐ray kVp and slice thickness until it reached a limit. Conversely, the contrasts of carbon and polymer markers showed little variations with the X‐ray kVp and slice thickness. In the kV range, if photoelectric interactions dominate, the materials with higher atomic number Z absorb more photons leading to significant contrast differences among materials of different Z. The photoelectric interaction probability is approximately proportional to $Z^{3}$. As kVp increases, more Compton interactions are involved between photons and materials. Since Compton interaction probability is proportional to mass electronic density and most of the tested materials have similar mass electronic density values (but with different physical densities), as kVp increases, the photon material interaction probability tends to become similar among materials of different Z per mass (in the Compton range) or approximately proportional to Z per thickness. On the other hand, since photoelectric interaction probability is proportional to $Z^{3}$, although increase of kVp increases the probability of Compton interaction, at a certain kVp range, that increase may be slower in high‐Z materials, such as gold, than the surrounding phantom material, causing there to be more photoelectric interactions in the high‐Z material than the surrounding materials and leading to an increase in the contrast. For the carbon and polymer markers, since their Z values are close to the surrounding material, the increase of kVp increases the Compton interactions in a similar rate for the markers and the medium, leading to a decrease in the contrast. This phenomenon indicates that the impact of fiducial markers on the CT image quality can be adjusted for the gold type by adjusting CT parameters, but not substantially for the carbon and polymer type of markers. When kV imaging‐based treatment verification equipment is available, polymer and carbon markers may be the preferred choice for target localization and patient treatment positioning verification because they produce fewer artifacts than gold markers on the simulation CT images but are still clearly identifiable on the kV verification images. If MV imaging will be the sole modality for positioning verification, including both linac‐based MV portal imaging and tomotherapy MVCT imaging, it may be necessary to use gold markers despite the artifacts they create on the simulation CT images, because the carbon and polymer markers cannot be clearly identified without any ambiguity.

Aside from affecting organ delineation during treatment planning and patient setup, the artifacts from fiducial markers can also cause perturbation of the dose distribution in the patient. One experimental study found that in a phantom embedded with a gold seed and irradiated with a 6 MV photon beam, there was about a 21% increase in dose 0.35 mm proximal to the gold seed and about a 22% decrease distal to the seed.^(21)^ A Monte Carlo‐based study found that the presence of a gold seed irradiated by a 6 or 18 MV photon beam in water affects the dose distribution at about 3 mm distance beyond both the upstream and downstream seed surface when compared to the relative dose profiles without the seed. When normalized to the dose at 5 mm above the isocenter, the relative doses upstream from the seed surface were found to be 1.64 for 6 MV and 1.56 for 18 MV photon beams parallel to the width of the seed.^(22)^ A high‐Z marker could be even more of a problem with proton treatments, where a fiducial marker in the path of the beam could cause a shift in the Bragg peak. Several studies have calculated the magnitude of dose perturbation for proton treatments as a function of marker material, implantation depth, and orientation with respect to the beam axis for various marker materials, including gold, carbon‐coated ceramic, stainless steel, and tantalum.^9^, ^13^, ^15^, ^20^ One Monte Carlo‐based study found a 5% dose increase upstream and a 2% decrease downstream for gold markers and 250 MeV protons.^(15)^ A study by Lim et al.^(16)^ showed that by mixing microscopic gold particles and human‐compatible polymers, one could create a fiducial marker for proton therapy that had good radiographic visibility, low distortion of the depth‐dose distribution, and few CT artifacts.

Metal artifact reduction (MAR) methods have been employed to improve CT image quality when gold marker‐introduced image artifacts are present^17^, ^18^ A study by Kassim et. al.^(17)^ showed using a MAR method on the reconstructed CT set allowed the position and orientation of the markers to be indentified more accurately during organ localization. The study also noted that the MAR method often substantially suppressed streak artifacts surrounding the metallic markers, allowing for better organ delineation during treatment planning. MAR software does not always result in improved image quality. A study by Liu et. al.^(19)^ noted that the MAR reconstruction algorithm improved CT image quality for patients with large metal orthopedic implants, but introduced blurring artifact when used on patients with small metal implants.

There are several limitations in the current study. First, images were only acquired and examined on a cubic phantom constructed from a bolus material. Therefore, the results of this study may be only applicable to soft tissue and not necessarily valid for cases where the fiducial markers are placed inside or near bone or lung. The near constant density bolus phantom allowed us to quantify the artifacts purely introduced by the fiducial markers and exclude effects from patient anatomy. Another limitation of the study is that the examinations were performed on digital images acquired by imaging modalities manufactured by a limited number of vendors. Since the quality of digital images may be very dependent on methods of signal detection and image reconstruction, the results may be different for similar types of imaging devices of different models. A clinic may want to perform their own similar study with the imaging modalities utilized in their department using several types of fiducial markers.

## V. CONCLUSIONS

Our data show the dependence of three selected fiducial marker materials on various imaging parameters and can serve as guidelines for marker type selection under a certain IGRT environments. It can also serve as guidelines for the improvement of imaging quality by adjusting certain imaging parameters when markers are present. It is recommended that this study be used as a starting point for future studies using patient data or a phantom more anatomical similar to a human. With the uniform bolus phantom used, we found that when kV imaging‐based treatment verification equipment is available, polymer and carbon markers may be the preferred choice for target localization and patient treatment positioning verification due to less image artifacts. If MV imaging is the sole modality used for positioning verification, it may be necessary to use gold markers despite the artifacts they create on the simulation CT images. In a clinic where mixed kV and MV‐based imaging schemes are used, additional evaluations may be needed, depending on the imaging energy levels available and specific techniques used, to identify an optimal combination with the chosen fiducial markers.
